# Examination of Vitreolenticular Interface in Relation to Different Phacoemulsification Parameters in Early and Late Postoperative Period

**DOI:** 10.3390/jcm13164855

**Published:** 2024-08-17

**Authors:** Ágnes Elekes, Péter Vámosi

**Affiliations:** 1 Department of Ophthalmology, Péterfy Sándor Hospital, 1076 Budapest, Hungary; 2 Doctoral School of Clinical Medicine, University of Debrecen, 4032 Debrecen, Hungary

**Keywords:** phacoemulsification, anterior segment optical coherence tomography, anterior vitreous detachment

## Abstract

**Background**: The surgical parameters of phacoemulsification can significantly impact the behavior of the anterior hyaloid membrane (AHM). **Methods**: In this prospective study, anterior segment optical coherence tomography was used to examine the attachment or detachment of the AHM of 82 eyes after uneventful phacoemulsification preoperatively and postoperatively over 1 year. The impacts of the capsulorhexis’ size, number of hydrodissections, nuclear sclerosis grade, cumulative dissipated energy (CDE), ultrasonic time, total surgical time, weakness of zonular fibers, presence of lens materials in Berger’s space (LM-BS), and fluid usage were investigated in relation to the behavior of the AHM. **Results**: A significant linear trend regarding anterior vitreous detachment (AVD) was observed in the presence of zonular weakness and high CDE at all postoperative times (*p* ≤ 0.024 and *p* ≤ 0.005, respectively). Similarly, AVD was observed at 1-month, 3-month, and 1-year follow-ups in cases of high nuclear sclerosis grades (*p* ≤ 0.044) and high fluid usage (*p* ≤ 0.021). A significant correlation was observed in the group of LM-BS as the zonular weakness value increased (OR: 0.085; 95% CI: 0.017 to 0.420; *p* = 0.002), and the fluid usage was also significantly higher (OR: 1.049; 95% CI: 1.003–1.096; *p* = 0.037). **Conclusions**: Zonular weakness, high CDE, a hard nucleus, and high fluid usage are risk factors for postoperative AVD.

## 1. Introduction

It is well known that phacoemulsification fundamentally changes the state of the lens and that the operation also affects the integrity of the whole eye. The most studied parts of the eye are the corneal endothelium and the corneal curvature. The former can be significantly damaged due to high flow rates and ultrasound energy or the insufficient use of viscoelastic material [1,2], while the latter is primarily influenced by the size of the wound, its location, its relation to the limbus, and the mechanical effects suffered during surgery [3,4]. Experienced surgeons know how to perform phacoemulsification while causing as little damage to the iris as possible. Since 1975, several publications have reported that posterior vitreous detachment (PVD) occurs much more often after cataract extraction [5,6,7] and that the chance of retinal detachment increases by several times after phacoemulsification, especially in highly myopic eyes [6,7,8]. Meanwhile, the vitreolenticular compartment and the behavior of the anterior hyaloid membrane (AHM) after phacoemulsification have only recently been studied. The behavior of Berger’s space (BS) (first described by Wieger [9]) during phacoemulsification was first reported by Tassignon and Dhubhghaill in 2016 [10]. Real-time intraoperative optical coherence tomography (iOCT) [10,11,12,13,14] and anterior segment optical coherence tomography (AS-OCT) [11,12,14,15,16,17,18] were used to visualize the posterior capsule, the AHM, and the possible change in the intervening BS during surgery [10,11,12,13,14]. It is now clear that phacoemulsification significantly impacts BS [10,11,12,13,14,15,16,17,18]. After phacoemulsification, the preoperatively existing BS, in most cases, enlarges after surgery, or the preoperatively attached AHM detaches from the posterior capsule in certain cases [12,14,15,16,17,18]. It has been proven that the age of the patient, the eye’s axial length, the presence of lens materials in BS (LM-BS), the weakness of zonular fibers, and corneal power affect the behavior of the AHM [12,13,14,15,16,18]. Data are available on how surgical parameters of phacoemulsification, such as the total surgical time, cumulative dissipated energy (CDE), ultrasonic time, mean longitudinal power, total aspiration time, fluid usage, infusion pressure, aspiration flow rate, bottle height, and vacuum, influence the development of BS [12,14,15,17,19]. However, some surgical parameters that can be assumed to affect the state of the vitreolenticular compartment have not been investigated. In this study, we additionally investigated the significance of the parameters of the nuclear sclerosis grade, hydrodissection, and capsulorhexis size. Previous studies showed that LM-BS and certain anatomical parameters were correlated. The relationships between LM-BS and some surgical parameters of phacoemulsification were also studied [10,11,12,13,14].

Until now, the vitreolenticular interface has been observed for a maximum term of 3 months. To the best of our knowledge, this is the first 1-year-long prospective report documenting the attachment or detachment of the AHM after and related to intraoperative parameters during phacoemulsification. AHM detachment will be referred to as anterior vitreous detachment or AVD hereon.

## 2. Materials and Methods

In this prospective non-randomized interventional study, we assessed the evolution of the retrolental space before and after cataract surgery using swept-source anterior segment optical coherence tomography(SS-AS-OCT). This study was conducted with approval from the Local Ethics Committee and per the Declaration of Helsinki.

### 2.1. Patients

We examined 82 eyes of 82 patients between July 2021 and July 2023 preoperatively and at 1-day, 1-month, 3-month, and 1-year follow-ups. Visual acuity, intraocular pressure, pupil dilation, and slit-lamp examination with fundus examination were performed at each check-up. All patients had preoperative Lens Opacity Classification System III (LOCSIII) grade 1–6 nuclear sclerosis diagnosed using a slit-lamp examination. SS-AS-OCT during pupil dilation was performed preoperatively and at each follow-up with Anterion (Heidelberg Engineering, Heidelberg, Germany). Eyes with corneal, iris, or lens malformations, phacodonesis, or poorly dilated pupils, and those that had previously undergone any eye surgery, were excluded from this study. Out of the initial 100 eyes, posterior capsular rupture occurred in 1 case, and 17 patients did not come for regular check-ups. These eyes were also excluded from this study.

### 2.2. Surgical Procedure

The operations were performed by a single experienced surgeon under topical anesthesia. A 2.7 mm clear corneal incision was placed in the temporal 180–0 degrees, followed by uneventful phacoemulsification and implantation of a one-piece intraocular lens (IOL) into the capsular bag. The Megatron S4 phaco system (Geuder AG, Heidelberg, Germany) was used. The stop-and-chop technique was performed with the following fluidic parameters: a flow rate of 25 mL/min, a maximal vacuum of 300 mmHg, a maximal ultrasound energy of 70%, linear cold flash mode, and an infusion bottle height of 70 cm. The following parameters were used during the irrigation–aspiration of the cortex material: a flow rate of 23 mL/min, a maximal vacuum of 540 mmHg, and an infusion bottle height of 70 cm.

### 2.3. Intraoperative Data Collection

The following data were recorded: the capsulorhexis size (with an accuracy of 0.5 mm), number of hydrodissections, CDE, ultrasonic time, total surgical time, LM-BS (yes or no), and fluid usage (with an accuracy of 20 mL). The weakness of zonular fibers was semi-quantitatively described and scored on a scale of 1 to 4. The following aspects were considered when setting the scores: scale 1 = tight zonules, i.e., no rebound effect during nuclear rotation, a stable anterior chamber depth during surgery, and no posterior capsule wrinkling during polishing; scale 2 = medium weak zonules, i.e., no rebound effect during nuclear rotation, a slight fluctuation in the chamber depth during surgery, and slight posterior capsule wrinkling during polishing; scale 3 = weak zonules, i.e., a rebound effect during nuclear rotation, a high fluctuation in the anterior chamber depth during surgery, and a highly wrinkled posterior capsule during polishing; and scale 4 = very weak zonules, i.e., the characteristics described at scale 3 were so severe that the surgeon considered capsular tension ring implantation to be necessary.

### 2.4. Image Acquisition

**Figure 1:**
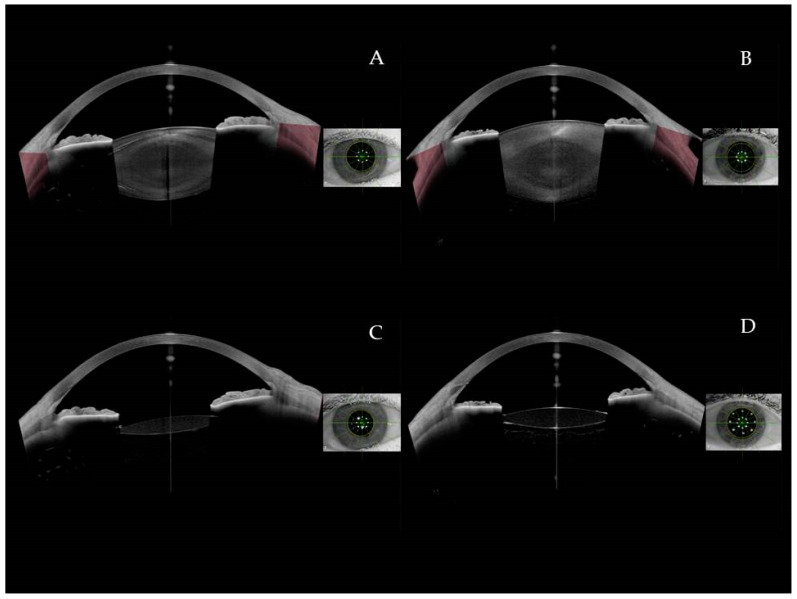
Anterior segment optical coherence tomography AS-SS-OCT images: preoperative images: without (**A**) visible anterior hyaloid detachment (AHD) and with visible AHD (**B**). Postoperative images: without (**C**) visible AHD and with visible AHD (**D**).

Image acquisition was performed per the method description created in our previous article [18]. Preoperatively, we performed an intraocular lens power calculation with the Cataract App of the Anterion device. We also captured eyes using the Metrics App. The Metrics App’s automatic mode creates multiple radial B-scans of up to 16.5 mm in length and 14.0 mm in depth using a 1300 nm wavelength. The images taken depicted the cornea, anterior chamber, chamber angle, iris, and lens, but the visibility of the retrolental space in phakic eyes was limited. The presence or absence of AHM was checked in the Imaging App. Deep and clear images of retrolental space could be obtained after turning off the Imaging App’s automatic mode in the manual setting by pushing the device closer to the eyes. We ensured that the hyperreflective line visible in the Metrics App’s mode was not just a vitreous fiber but was actually the AHM by choosing the most appropriate dense pattern in the Imaging App in manual mode. Postoperatively, we only used the Metrics App in automatic mode in all of the follow-ups, and the AHM was visible behind the narrow IOL. Image Acquisition is illustrated by Figure 1.

### 2.5. Statistical Analysis

The quantitative variables given included the valid N, mean, standard deviation, minimum, 25th percentile, median, 75th percentile, and maximum values, while the case qualitative variables included the number of cases and the percentage.

The Fisher test (less than 5 observed values) or the Pearson chi-square test (more than 5 observed values) was used for the qualitative variables. The linear association was also examined (linear-by-linear association) for the ordinal qualitative variables. Binary logistic regression was tested for LM-BS before backward Wald feature selection was applied. The results of the Hosmer–Lemeshow test were then given.

The Kolmogorov–Smirnov test was used to examine the deviation in the variables from their normal distribution. Since the distribution of all of the variables, except for age, differed from a normal distribution, non-parametric versions of the tests were applied. An independent *t*-test (two groups) or one-way ANOVA test (>two groups) was used to test the differences between individual groups. The Bonferroni correction was used to modify the *p*-value for multiple comparisons.

The limit of statistical significance was *p* < 0.05.

## 3. Results

In total, 82 eyes of 82 patients were included in the analysis, including 21 (25.6%) men and 61 (74.4%) women (mean age: 70.9 years). The average AL was 23.43 mm. The AHM conditions for the different follow-up times are summarized in Table 1.

The distribution of the values of the capsulorhexis size, number of hydrodissections, weakness of zonular fibers, and LM-BS (yes or no) are shown in Table 2.

Below, the results are discussed concerning the AHM situation. The significant correlations are discussed separately for the follow-up dates, and the LM-BS is described in a separate paragraph regarding the intraoperative surgical parameters.

Postoperative 1-day follow-up: A significant linear trend was observed between the postoperative 1-day AVD and the increasing degree of zonular weakness (*p* = 0.023). The CDE was significantly higher in the 1-day postop AVD group (*t*-test: *p* = 0.005).

One-month follow-up: A significant linear trend was observed between the presence of AVD at one-month follow-up and high-grade nuclear sclerosis (*p* = 0.011), respectively, with the increase in intraoperative zonular weakness (*p* = 0.001). The CDE (*t*-test: *p* = 0.001) and higher fluid usage (*t*-test: *p* = 0.012) were significantly higher in the 1-month postop AVD group. A significant correlation was observed between the 1-month AVD presence and the LM-BS intraoperatively (*p* = 0.026).

Three-month follow-up: A significant linear trend was observed between the visible AVD at the three-month follow-up and high-grade nuclear sclerosis (*p* = 0.014), and a significant linear trend was observed with increasing zonular weakness (*p* = 0.004). A correlation was seen between AVD and a higher CDE (*t*-test: 0.001), high total surgical time (*t*-test: 0.028), and high fluid usage (*t*-test: 0.021).

One-year follow-up: A significant linear trend was observed between AVD at the one-year follow-up and high-grade nuclear sclerosis (*p* = 0.015), and a significant linear trend was observed with increasing zonular weakness (*p* = 0.024). A higher CDE (*t*-test: 0.002) and high fluid usage (*t*-test: 0.014) correlated with 1-year AVD.

The capsulorhexis size, number of hydrodissections, and ultrasonic time did not show a significant correlation with AVD at any time point.

The associations between the intraoperative parameters and the presence of LM-BS were as follows.

Intraoperative LM-BS was found in 12 eyes (14.6%). Among the examined surgical parameters, zonular weakness and fluid usage showed a statistically significant correlation with LM-BS using multiplex regression. Zonular weakness was significantly correlated with LM-BS status, i.e., the looser the zonular fibers, the more likely LM-BS was to occur (odds ratio: 0.085 (95% confidence interval: 0.017–0.420; *p* = 0.002)). Fluid usage was significantly higher in those with LM-BS (odds ratio: 1.049 (95% confidence interval: 1.003–1.096; *p* = 0.037)). The nuclear sclerosis grade, CDE, total surgical time, capsulorhexis size, number of hydrodissections, and ultrasonic time did not show significant correlations with the intraoperative presence of LM-BS.

## 4. Discussion

In 1986, Weidle described a method of visualizing the BS in vivo by injecting an ophthalmic viscosurgical device to protect the AHM during posterior capsulotomy [20]. Tassignon et al., who routinely performed bag-in-the-lens implantation, developed a technique for sparing the AHM during posterior capsulorhexis [21]. In 2016, Tassignon and Dhubhghaill used iOCT to visualize the AHM and the changes in BS during phacoemulsification. They hypothesized that BS is a true anatomical space with a true anatomical purpose that must be protected [10]. In 2017, Masuda et al. suggested the use of an irrigation dynamic pressure-assisted hydrodissection technique that prevents high-pressure hydrodissection-related complications, such as capsular block syndrome and tears in the AHM during cataract surgery [22]. Scarfone et al. proved that the high infusion pressure used during phacoemulsification detrimentally affects the AHM barrier, which can be prevented using active fluidics and active sentry [17]. These examples show that it is worthwhile to investigate, as broadly as possible, which phacoemulsification parameters are predisposed to AVD.

In our study, a significant linear trend was observed between the visible AVD and zonular weakness (*p* ≤ 0.024) at all follow-up times. A high CDE correlated with AVD at all follow-up times (*t*-test: *p* ≤ 0.005). Similarly, AVD was observed at 1-month, 3-month, and 1-year follow-ups in cases of high-grade nuclear sclerosis (*p* ≤ 0.044) and high fluid usage (*p* ≤ 0.021). Conversely, the impact of the capsulorhexis size, number of hydrodissections, and total surgical time showed no significant associations with AVD. Several authors have investigated the effects of various phacoemulsification parameters on postoperative AVD [12,14,15,16,17]. These studies’ results are summarized in Table 3.

Our experience agrees with the relevant literature suggesting that zonular weakness [12], higher-than-average ultrasound use [12,14,15], high-pressure infusion [15,17], and higher CDE [14] predispose the patient to AVD. Previous comparisons did not find significant correlations between AHM detachment and high-grade nuclear sclerosis and high fluid usage [14,16]; however, we observed that both parameters were significantly correlated with AHM detachment for most of the follow-up times. This issue might require further investigation.

Furthermore, we observed that the AHM detachments related to the intraoperative risk factors did not change during the one-year follow-up period. Vael et al. identified age as a risk factor for AVD. Their model determined that the odds for AVD increased by 5.3% for each year of patients’ age in the group of cataract patients [15]. It would be worth investigating whether this finding is also valid for the post-phacoemulsification population. This issue requires a follow-up period of considerably longer than one year.

According to our current knowledge, it remains unclear how important it is to preserve the integrity of the vitreolenticular interface, when possible, during phacoemulsification. We agree with Tassignon’s theory that the fluid that collects within the 8 to 9 mm annular Wieger’s ligament functions like a synovium, lubricating the shape change of the lens [10]. A non-detached anterior hylaoid membrane can have a role in stabilizing postoperative refraction. Mori reported that the postoperative refractive prediction was less accurate in eyes with visible AHM after phacoemulsification [16].

Meanwhile, the disruption of the vitreolenticular compartment allows for the diffusion of different microbes and inflammatory mediators through zonules into BS, consequently increasing the risk of vitritis, macular edema, and endophthalmitis. According to various sources, 2.0–25.0% of bacteria are cultured after phacoemulsification from the anterior chamber, although the basic rules of perioperative asepsis and antisepsis were strictly observed [23,24,25]. Hence, we think it is important to know exactly the surgical parameters that influence the state of BS. This knowledge will make it easier to use a surgical technique that, if possible, preserves the integrity of the vitreolenticular compartment. Surgeons should carefully adjust the ratio between the bottle height and flow rate to achieve a balanced state, which may enhance the stability of fluid circulation and safety during surgery. Therefore, CDE and fluid usage should be appropriately reduced in cataract patients with weakened zonular fibers, such as those with high myopia and pseudoexfoliation syndrome, to mitigate the damage to zonules and decrease AVD incidence.

The LM-BS concept has been used in the literature when the lens material has been visualized with different methods during cataract surgery. This may include the modern iOCT, but the LM-BS also has a characteristic image with an operating microscope [10,11,12,13,14]. The LM-BS has also been postoperatively assessed using AS-OCT [11,14]. Our findings agree with those of Anisimova [11] and Lin [14], indicating that phacoemulsification can result in zonular dehiscence, AHM detachment, and Wieger’s ligament injury, which can lead to lens materials entering BS.

In our study, additional risk factors for LM-BS were found. A significant correlation was observed in the group of LM-BS as the zonular weakness value increased. Fluid usage was also significantly higher in the LM-BS group. We believe that if LM-BS is intraoperatively detected, it most likely indicates a breakdown of the integrity of the vitreolenticular anatomy. As such, it may be worthwhile to implant a capsular tension ring in such cases. In this way, the lens capsule could be stabilized; therefore, the postoperative refraction could be theoretically stabilized more quickly. This assumption should be further investigated.

This study had several limitations. Zonular weakness was semi-quantitatively described. Excluding eyes with pre-existing problems and individuals who did not complete follow-ups may have introduced selection bias. Including a larger and more diverse sample to enhance the generalizability of the findings could improve this study.

## 5. Conclusions

In summary, this is the first prospective report documenting the impact of the surgical parameters of phacoemulsification on the behavior of the AHM over 1 year. The effects of the capsulorhexis’ size, number of hydrodissections, nuclear sclerosis grade, CDE, ultrasonic time, total surgical time, weakness of zonular fibers, presence of LM-BS, and fluid usage were investigated in relation to the behavior of the AHM using SS-AS-OCT. Additionally, the relationships between LM-BS and certain eye parameters, as well as certain surgical parameters of phacoemulsification, were studied. A significant linear trend was observed between the visible AHM detachment and zonular weakness at all follow-up times. Furthermore, a high CDE correlated with the AHM detachment group at all follow-up times. Similarly, AHM detachment was observed at 1-month, 3-month, and 1-year follow-ups in cases of high-grade nuclear sclerosis and high fluid usage. Zonular weakness and high fluid usage seem to be risk factors for the presence of LM-BS.

## Figures and Tables

**Table 1:** Anterior hyaloid membrane (AHM) conditions for the different follow-up times.

	Preop		Postop 1 Day		Postop 1 Month		Postop 3 Months		Postop 6 Months	
Attached AHM	58	70.7%	37	45.1%	40	48.8%	35	42.7%	36	43.9%
AVD	24	29.3%	45	54.9%	42	51.2%	47	57.3%	46	56.1%

**Table 2:** Distribution of values of capsulorhexis size, number of hydrodissections, weakness of zonular fibers, and LM-BS. Abbreviations: LM-BS = lens material in Berger’s space.

Capsulorhexis Size					
Size (mm)	4	4.5	5	5.5	6
Number of cases	6	24	41	9	1
Percentage	7.4%	29.6%	50.6%	11.1%	1.2%
Number of Hydrodissections					
Number of hydrodissections	1	2	3	4	6
Number of cases	48	24	5	4	1
Percentage	58.5%	29.3%	6.1%	4.9%	1.2%
Weakness of Zonular Fibers					
Level of weakness	Tight	Medium	Weak	Very weak	
Number of cases	14	53	11	4	
Percentage	17.1%	64.6%	13.4%	4.9%	
Lens Material in BS During Surgery					
	Yes	No			
Number of cases	12	70			
Percentage	14.6%	85.4%			

**Table 3:** Effects of various parameters of phacoemulsification and cataract eye characteristics on the behavior of postoperative vitreolenticular compartment studied by previous authors. Yes: visible Berger’s space/ anterior hyaloid membrane (BS/AHM) or postoperative anterior vitreous detachment (AVD). No: invisible BS/AHM or no postoperative AVD. Abbreviations: CDE = cumulative dissipated energy; US time = ultrasonic time; surg. time = total surgical time; asp. time = aspiration time; fluid us. = fluid usage; irr. pr. = irrigation pressure; inf. pr. = infusion pressure; zon. weak. = zonular weakness; NCG = nuclear sclerosis grade; LT = lens thickness; AL = axial length; CP = corneal power; CC = corneal cylinder.

	CDE	US Time	Surg. Time	Asp. Time	Fluid Us.	Irr. Pr.	Inf. Pr.	Zon. Weak.	NCG	LT	AL	CP	CC
Lin et al. [12]	Yes	-	-	-	-	-	-	Yes	-	-	-	-	-
Lin et al. [14]	Yes	No	-	Yes	No	-	-	-	No	-	-	-	-
Zhang et al. [15]	-	-	Yes	-	-	Yes	-	-	-	No	No	-	-
Mori et al. [16]	-	-	-	-	-	-	-	-	No	-	Yes	Yes	No
Scarfone et al. [17]	-	-	-	-	-	-	Yes	-	-	-	-	-	-

## Data Availability

The data will be made available upon request from the corresponding author.
